# Lipoprotein(a) in the statin era: from risk marker to therapeutic target

**DOI:** 10.3389/fphar.2026.1938864

**Published:** 2026-09-16

**Authors:** Rongshen Hong, Yuhua Liu, Xiaochun Li, Hongxiao Liu, Dongyang Xie

**Affiliations:** 1 First Clinical Medical College, Gannan Medical University, Ganzhou, China; 2 The First Affiliated Hospital of Gannan Medical University, Ganzhou, China

**Keywords:** antisense oligonucleotide, cardiovascular outcomes trial, lipoprotein(a), residual cardiovascular risk, RNA therapeutics, screening, siRNA

## Abstract

Lipoprotein(a) [Lp(a)] is a predominantly genetically determined lipoprotein whose concentration is generally stable over time, although clinically relevant within-person variation can occur. Elevated concentrations affect about one in five people. It has emerged as one of the most important causal contributors to the residual cardiovascular risk that persists despite effective LDL-cholesterol lowering in the statin era, and it is also causally implicated in calcific aortic valve stenosis. For decades Lp(a) was measurable yet effectively untreatable, because conventional lipid-lowering therapies do not reduce it to a clinically meaningful degree. Two practical obstacles further constrain its clinical use. Analytical standardization is incomplete: mass and molar units are not interchangeable, and some immunoassays are biased by apolipoprotein(a) isoform size. Real-world screening rates also remain very low, despite a guideline consensus on once-in-a-lifetime testing. This picture is now changing rapidly. RNA-targeted agents, comprising antisense oligonucleotides and small interfering RNAs, together with a first oral small-molecule inhibitor, lower Lp(a) by about 65% to more than 95%, differing in mechanism, route of administration, dosing interval and durability. Two questions remain open: how large an absolute reduction is needed for clinical benefit (the threshold hypothesis), and whether lowering Lp(a) reduces major adverse cardiovascular events at all. Several cardiovascular outcomes trials are now testing these questions directly. They include Lp(a)HORIZON, OCEAN(a)-Outcomes, and ACCLAIM-Lp(a), together with a dedicated aortic-valve trial, and the first readouts are anticipated. Framing Lp(a) as the residual cardiovascular risk target of the statin era, this review integrates measurement, screening, risk stratification, current management, and emerging targeted therapies into a single clinical and pharmacological roadmap for the outcomes-trial era.

## 1 Introduction

Lowering low-density lipoprotein cholesterol (LDL-C) with statins reduces major vascular events by about one-fifth for each 1 mmol/L reduction (Baigent et al., 2005; Baigen et al., 2010). More intensive statin regimens (Cannon et al., 2004) and the addition of non-statin agents (Cannon et al., 2015) extend this benefit further. Yet patients who reach very low LDL-C on modern therapy still have cardiovascular events. Even trials that drove LDL-C below 30 mg/dL with PCSK9 inhibition left a considerable burden of risk untouched (Sabatine et al., 2017; Schwartz et al., 2018). Defining and treating the drivers of this residual risk has therefore become a central task of preventive cardiology.

Lipoprotein(a) [Lp(a)] is now recognized as one of the most important of these drivers. The particle has an LDL-like core whose apolipoprotein B is covalently linked to apolipoprotein(a), a glycoprotein structurally homologous to plasminogen. This dual identity connects Lp(a) to both atherogenesis and thrombosis (McLean et al., 1987). Plasma concentrations are roughly 80%–90% determined by the *LPA* locus and are generally stable over time, making Lp(a) a predominantly inherited, lifelong exposure, although clinically relevant within-person variation may occur (Boerwinkle et al., 1992). Large prospective cohorts, genome-wide association studies, and Mendelian randomization analyses all point to Lp(a) as an independent, causal contributor to atherosclerotic cardiovascular disease (ASCVD) and to calcific aortic valve stenosis (Clarke et al., 2009; Erqou et al., 2009; Thanassoulis et al., 2013). About one in five people carries concentrations high enough to raise cardiovascular risk. High Lp(a) predicts events even in statin-treated patients who have met guideline LDL-C targets (Willeit et al., 2018).

For decades, Lp(a) held a difficult position: causally implicated, yet actionable only indirectly. Conventional lipid-lowering drugs have little useful effect on its concentration, and statins do not lower it and on average increase it modestly, although individual responses are heterogeneous (Tsimikas et al., 2020a). Two practical obstacles have deepened this therapeutic gap. First, measurement remains poorly standardized. Some mass-based immunoassays are biased by apolipoprotein(a) isoform size and cannot be reliably converted to molar units, so a single reported value may mislead (Marcovina et al., 2022). Second, real-world testing rates have stayed very low, even though international guidelines now advise measuring Lp(a) at least once in every adult (Kronenberg et al., 2022; Koschinsky et al., 2024; Bhatia et al., 2023). The result is a risk factor that is common, causal, and easily overlooked.

This picture is changing quickly. RNA-targeted therapies (antisense oligonucleotides and small interfering RNAs), together with a first oral small-molecule inhibitor, now lower Lp(a) by about 65% to more than 95% in early-phase trials (Tsimikas et al., 2020b; O'Donoghue et al., 2022). Whether such reductions translate into fewer cardiovascular events remains unproven. Genetic modeling suggests that a large absolute reduction in Lp(a) may be needed before clinical benefit emerges, which helps explain why earlier, weaker interventions failed (Lamina et al., 2019). Several cardiovascular outcomes trials are now testing this hypothesis directly, and their first readouts are anticipated.

Existing reviews have generally emphasized either the pharmacology of these emerging agents or isolated clinical recommendations. We take a different approach. We frame Lp(a) as the residual cardiovascular risk target of the statin era and follow it through a single clinical and pharmacological loop: from measurement and screening, through risk stratification and current management, to the targeted therapies and outcomes trials that may soon close it. Two questions stay in view throughout: how reliably Lp(a) can be measured and interpreted in everyday practice, and how much lowering will be needed to change outcomes. Holding both in view lets each advance be placed in a coherent, actionable framework for clinicians. The literature search underpinning this review was current to 8 August 2026.

## 2 Biology, pathophysiology, and causal evidence

### 2.1 Structure and the apolipoprotein(a) isoform

Lipoprotein(a) is built on a low-density lipoprotein (LDL)-like core: a cholesterol-rich particle carrying a single molecule of apolipoprotein B-100. To this core the distinctive glycoprotein apolipoprotein(a) [apo(a)] is covalently attached through a single disulfide bond. The cloning of apo(a) revealed that it is closely homologous to plasminogen, with multiple copies of a plasminogen-like kringle IV domain, one kringle V domain, and an inactive protease-like region (McLean et al., 1987). This structural duality, an atherogenic lipoprotein fused to a fibrinolysis-related protein, grounds the mechanistic account that follows and sets Lp(a) apart from every other atherogenic particle (Figure 1a).

**FIGURE 1:**
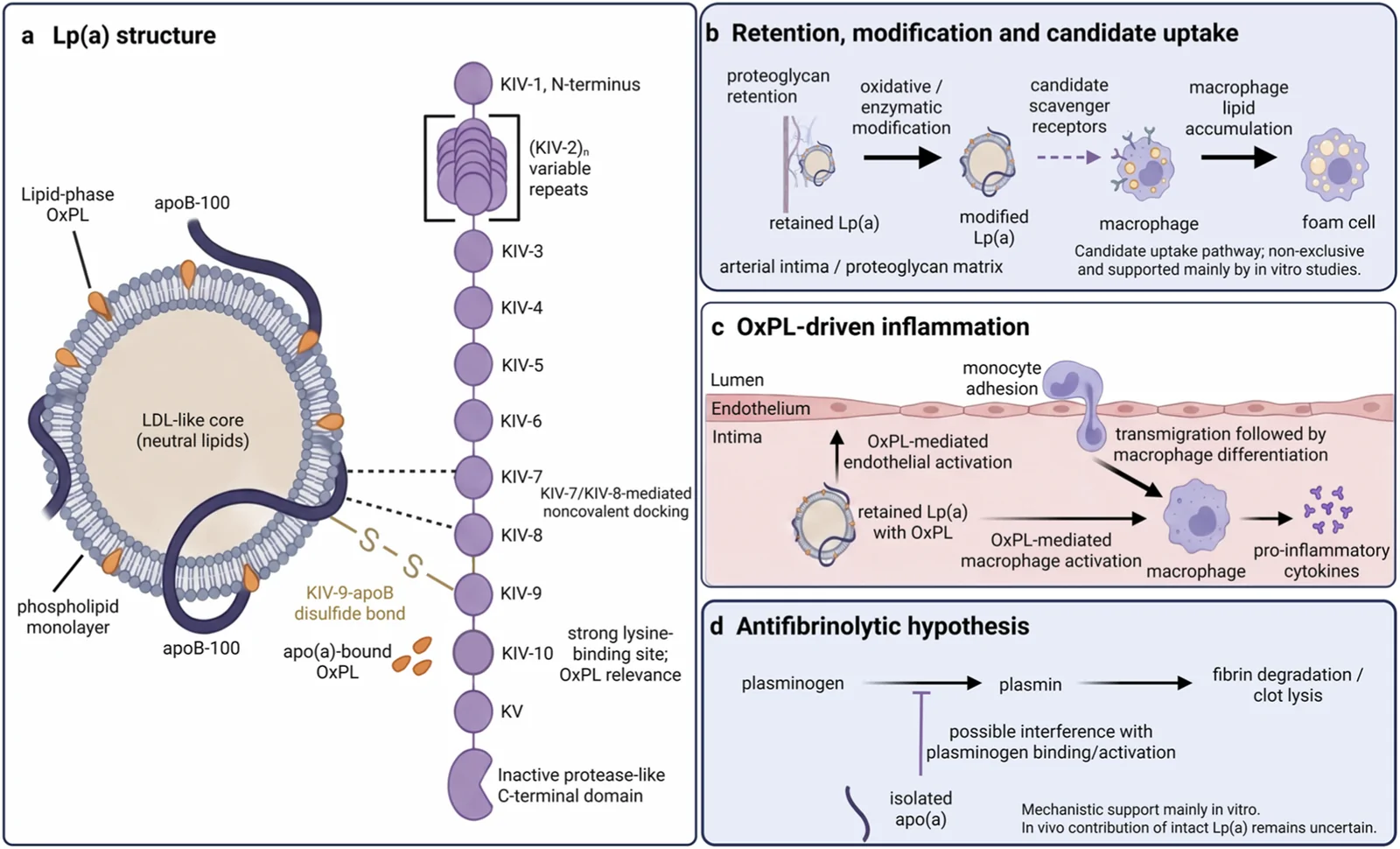
Structure and proposed pathophysiological pathways of lipoprotein(a). **(a)** Lp(a) consists of an LDL-like particle containing one apolipoprotein B-100 (apoB-100) molecule linked to apolipoprotein(a) [apo(a)] by a disulfide bond involving KIV-9. A preceding noncovalent docking interaction, attributed mainly to KIV-7 and KIV-8, is indicated by dashed connector lines. Apo(a) comprises KIV-1 at the N-terminus, a variable number of KIV-2 repeats, single copies of KIV-3 to KIV-10, kringle V (KV), and an inactive protease-like C-terminal domain. KIV-10 contains a strong lysine-binding site and is identified separately for its relevance to OxPL biology. Oxidized phospholipids (OxPL) are depicted in two pools: lipid-phase OxPL and apo(a)-bound OxPL. The precise attachment site and chemistry of apo(a)-bound OxPL have not been definitively established. The diagram is schematic and not drawn to scale. Panel **(a)** was redrawn on the basis of primary structural studies (McLean et al., 1987; Leibundgut et al., 2013; Haibach et al., 1998; Koschinsky et al., 1993; Becker et al., 2004; Rahman et al., 2002). **(b)** Retention, modification and candidate uptake: Lp(a) is retained by proteoglycans in the arterial intima and may undergo oxidative or enzymatic modification. Uptake through candidate scavenger receptors is shown as a dashed arrow because the receptors are non-exclusive, no single Lp(a)-specific receptor has been established, and the pathway is supported mainly by *in vitro* studies. Macrophage lipid accumulation and foam-cell formation are shown as subsequent cellular states. **(c)** OxPL-driven inflammation: OxPL associated with retained Lp(a) promote endothelial activation and monocyte adhesion. Following transmigration and macrophage differentiation, OxPL-mediated macrophage activation is associated with the release of pro-inflammatory cytokines. **(d)** Antifibrinolytic hypothesis: isolated apo(a) may interfere with plasminogen binding and activation, a mechanism supported mainly by *in vitro* evidence. Whether intact Lp(a) impairs fibrinolysis *in vivo* remains uncertain. Solid arrows indicate supported processes, dashed arrows indicate candidate or hypothesized pathways, and T-bars indicate possible interference.

The kringle IV region is not uniform. Its type-2 subunit is encoded by a variable number of tandem repeats in the *LPA* gene, so apo(a) exists as more than forty size isoforms that differ in the number of kringle IV-2 copies. Isoform size is inversely related to the rate of apo(a) synthesis, and so to plasma Lp(a) concentration. People carrying smaller isoforms tend to have higher circulating levels. Because this genetic architecture is dominant, the *LPA* locus explains more than 90% of the interindividual variation in Lp(a). Concentrations are set largely at birth and stay stable across the lifespan (Boerwinkle et al., 1992). Mendelian randomization analyses further show that both a small isoform size and a high measured concentration contribute independently to cardiovascular risk, so neither the genetically inferred nor the directly measured trait fully captures the other (Saleheen et al., 2017). Together these features, covalent apo(a)-apoB linkage, kringle-driven isoform heterogeneity, and strong genetic determination, frame Lp(a) as a predominantly inherited, lifelong exposure. They explain why it behaves so differently from the LDL-C that responds to diet and statins.

Although genetic factors account for most interindividual variation in Lp(a), circulating concentrations are not biologically immutable. Repeat-measurement studies demonstrate high overall within-person stability but clinically relevant variability, particularly at concentrations between 75 and 189 nmol/L (Ghouse et al., 2025). Non-genetic modifiers include kidney and liver disease, thyroid and sex-hormone states, and selected medications (Enkhmaa and Berglund, 2022). Dietary effects are heterogeneous and generally modest relative to genetically driven differences; physical-activity findings are inconsistent, and reductions reported after low-carbohydrate diets or bariatric surgery do not yet establish a broadly applicable lifestyle-based Lp(a)-lowering strategy (Enkhmaa and Berglund, 2022). The clinical significance of these changes remains uncertain.

### 2.2 Pathophysiology: one particle, three converging mechanisms

The pathogenicity of Lp(a) is best understood as three linked mechanisms carried on one particle. The first is pro-atherogenic. Like LDL, Lp(a) delivers apoB-bearing cholesterol into the arterial intima, where it is retained and taken up by macrophages. Unlike LDL, it also carries a disproportionate load of oxidized phospholipids (OxPL). These OxPL bind preferentially, and in part covalently, to apo(a), making Lp(a) the principal plasma carrier of these bioactive lipids (Tsimikas et al., 2005; Leibundgut et al., 2013). This coupling gives Lp(a) atherogenic potency beyond its cholesterol content alone (Figures 1b–d).

The second mechanism is pro-inflammatory and follows directly from the first. OxPL are recognized by innate immune receptors and drive monocyte activation and endothelial dysfunction. This is not merely an *in-vitro* observation. Positron-emission tomography imaging shows that people with high Lp(a) have measurably increased arterial-wall inflammation and monocyte trafficking, a finding that bridges the biochemistry of OxPL and the biology of plaque progression *in vivo* (van der Valk et al., 2016).

The third mechanism follows from the plasminogen homology of apo(a) and is pro-thrombotic. Because apo(a) resembles plasminogen but lacks fibrinolytic activity, it can interfere with plasminogen binding and activation. This may impair clot lysis and promote a prothrombotic state at sites of vascular injury (McLean et al., 1987; Boffa and Koschinsky, 2016). The *in-vivo* weight of this antifibrinolytic effect is less firmly quantified than the atherogenic and inflammatory pathways. It should therefore be regarded as biologically plausible and mechanistically supported, not as a definitively established driver of clinical events. Taken together, and across biochemical, imaging, and epidemiological evidence, these three mechanisms present Lp(a) as a particle that acts on several pathways at once, along both the atherosclerotic and the thrombotic axis of cardiovascular disease (No et al., 2016; Tsimikas, 2017).

In addition to its pathogenic effects, Lp(a) has been proposed to participate in wound healing and tissue repair, potentially through its interactions with fibrin and oxidized phospholipids (Kostakou et al., 2017). However, this putative physiological role remains incompletely defined and does not establish that elevated circulating Lp(a) is clinically beneficial.

### 2.3 Epidemiology, dose–response, and the causal chain

Mechanistic plausibility alone would not justify the clinical attention Lp(a) now receives. The decisive evidence is epidemiological and, above all, genetic. In the general population Lp(a) concentrations are highly skewed, and levels differ substantially by genetic ancestry, so both the distribution and the risk it confers vary across populations (Satterfield et al., 2021). Across this distribution the association with disease is graded and continuous. A collaborative meta-analysis of thirty-six prospective studies established an independent, continuous relationship between Lp(a) and both coronary heart disease and ischemic stroke (Erqou et al., 2009). Large population cohorts showed a stepwise increase in myocardial-infarction risk without an obvious lower threshold (Kamstrup et al., 2008). Recent biobank-scale data have sharpened this picture, describing an approximately linear dose–response with atherosclerotic cardiovascular disease and supporting the clinically used high-risk cut-points at the upper end of the distribution (Patel et al., 2021).

Association alone cannot establish causation, and here the lifelong genetic determination of Lp(a) becomes an analytical advantage. Because *LPA* variants randomize individuals to higher or lower Lp(a) from birth, they permit Mendelian randomization, a natural experiment largely free of confounding and reverse causation. Genetic studies show that *LPA* variants which raise Lp(a) also raise the risk of coronary disease. Formal Mendelian randomization has confirmed that genetically elevated Lp(a) is causally related to myocardial infarction (Clarke et al., 2009; Kamstrup et al., 2009). The same causal logic extends beyond the coronary bed. High Lp(a) is causally associated with calcific aortic-valve disease, a link first established genetically and then confirmed prospectively, with high Lp(a) predicting incident aortic stenosis (Thanassoulis et al., 2013; Kamstrup et al., 2014). Broader analyses have linked elevated Lp(a) to ischemic stroke and to increased cardiovascular and all-cause mortality, widening its clinical reach across the vascular system (Langsted et al., 2019a; Langsted et al., 2019b).

The magnitude of this causal effect may not be uniform across individuals. Analyses of the Multi-Ethnic Study of Atherosclerosis have reported that the association between Lp(a) and incident cardiovascular events is attenuated in participants with low systemic inflammation, indexed by high-sensitivity C-reactive protein, and in those with a favorable waist-to-hip ratio (Zhang et al., 2021; Ahmad et al., 2025). Elevated Lp(a) has likewise been described as prevalent among healthy centenarians (Thillet et al., 1998). These observations raise questions about effect modification rather than about causality itself. They derive from subgroups of a single primary-prevention cohort, in which low event rates limit statistical power, and the centenarian data are subject to survivor bias by construction, since survival to extreme age selects for individuals in whom elevated Lp(a) has not proved fatal; the assays available when those cohorts were characterized were also isoform-sensitive and not standardized against a common reference. A more fundamental consideration is that the absolute excess risk conferred by any given relative risk depends on background risk, so a smaller absolute effect in a metabolically healthy individual is expected even where the underlying causal effect is undiminished. Contemporary guidelines accordingly treat elevated Lp(a) as an independent risk-enhancing factor across the population rather than as a finding whose interpretation is conditional on metabolic phenotype, and Section 4 addresses Lp(a) as one component of a total risk burden that warrants comprehensive management.

This body of genetic evidence does more than confirm causality. It begins to quantify it, and in doing so it points ahead to the central therapeutic question of this review. Comparative Mendelian analyses suggest that the absolute reduction in Lp(a) needed to match the coronary benefit of lowering LDL-C by 1 mmol/L is large: published Mendelian estimates range from 65.7 to 101.5 mg/dL (Lamina et al., 2019; Burgess et al., 2018), differing according to the Lp(a) concentration estimates and assay architecture from which the underlying genetic associations were derived. In other words, the same genetics that prove Lp(a) is causal also imply that only a large reduction is likely to translate into clinical benefit. This is the threshold problem that recurs in the later sections. For the practicing cardiologist, the message of this section is therefore twofold. Lp(a) is a causal, lifelong, and mechanistically coherent contributor to atherosclerotic and valvular disease. Its effect is also steep enough that acting on it will demand both accurate measurement and large-magnitude lowering. The first requirement, turning a causal risk factor into a reliable clinical measurement, is the subject of the next section.

## 3 Measurement, screening, and risk stratification

Translating a causal risk factor into a clinical tool requires passing three gates in sequence. The value must be measured accurately, the measurement must actually be ordered, and the result must be interpreted correctly. For lipoprotein(a) each gate is fractured. Analytical standardization remains incomplete, so a single sample can yield discordant values. Guideline recommendations now favor near-universal testing, yet real-world measurement remains rare. And even when a value is obtained, its meaning depends on cut-points, intermediate ranges, and clinical context, not on any single number. These three gaps, more than any biological uncertainty, explain why a causal and lifelong exposure is still so unevenly used in practice. This section follows the same sequence and treats measurement standardization and the screening gap as the two obstacles that most directly limit clinical adoption (Figure 2).

**FIGURE 2:**
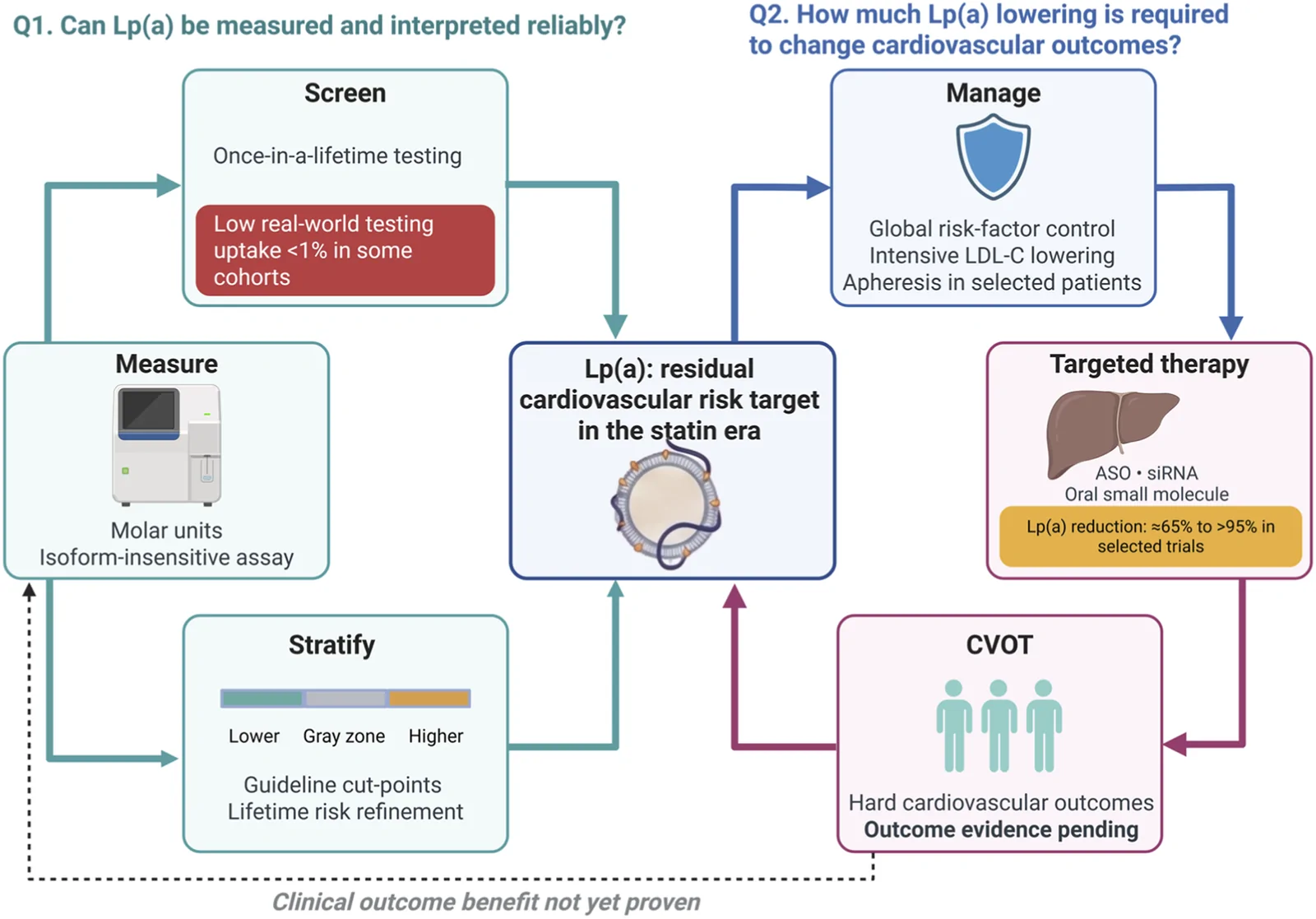
Lipoprotein(a) as the residual cardiovascular risk target of the statin era: a single clinical and pharmacological loop. The review is organized around two questions. Q1 (can Lp(a) be measured and interpreted reliably?) spans accurate measurement (assays insensitive to apo(a) isoform size, with results reported in molar units; isoform independence is determined by antibody epitope rather than by reporting unit), once-in-a-lifetime screening, and risk stratification (cut-points, the intermediate “gray zone” and special populations). Q2 (how much lowering is required to change outcomes?) runs from current management (global risk-factor and LDL-C reduction, selective lipoprotein apheresis) through targeted therapies (antisense oligonucleotides, siRNA and an oral small molecule; ∼65% to >95% Lp(a) lowering) to cardiovascular outcomes trials (CVOTs). The dashed arrow denotes that a cardiovascular outcome benefit of Lp(a)-specific lowering is not yet proven.

### 3.1 Analytical standardization: measuring accurately

The central analytical problem is that Lp(a) results are not interchangeable between assays, and the discrepancy comes from the isoform heterogeneity described earlier. What determines whether an assay is sensitive to apolipoprotein(a) size is the epitope its antibodies recognize, not the unit in which it reports. Antibodies directed at epitopes within the repetitive kringle IV type-2 region generate signal in proportion to the number of repeats a particle carries rather than to the number of particles, so such assays overestimate concentration when apolipoprotein(a) is larger than the calibrator and underestimate it when apolipoprotein(a) is smaller. This was shown directly in a comparison of three assays sharing a single capture antibody, in which only the assay whose detection antibody recognized the type-2 repeats displayed size-dependent bias (Marcovina et al., 1995). An assay can avoid that bias by reading an epitope present in a single copy: one monoclonal antibody ELISA, whose detection antibody binds a single-copy sequence in kringle IV-9, shows bias essentially independent of apolipoprotein(a) size across samples of known isoform size (Marcovina et al., 2022). A reference measurement procedure based on liquid chromatography–mass spectrometry approaches the problem differently again, quantifying apolipoprotein(a) through proteotypic peptides that lie outside the variable repeat, and is intended to anchor a future traceability chain rather than to serve as a routine clinical method (Ruhaak et al., 2023). This procedure has since been endorsed by the International Federation of Clinical Chemistry and Laboratory Medicine, and analytical validation of a semi-automated implementation has improved its precision across the measuring interval (van Neer et al., 2026). Commutable, ISO 15194-compliant serum-based secondary reference materials, value-assigned by transfer from the former WHO/IFCC reference material SRM 2B, are now available, and a network of three calibration laboratories operates the harmonized procedure, although full SI traceability has not yet been achieved (Cobbaert et al., 2026). Despite these advances, legacy mg/dL assays and fixed conversion factors remain in wide use. No single factor converts mass to molar units reliably across the range of isoforms, so the same patient can fall on different sides of a clinical threshold depending only on the assay used. Scientific statements have accordingly identified assay standardization as an unresolved barrier to the routine clinical use of Lp(a) (Reyes-Soffer et al., 2022).

### 3.2 Who and when to test: a converging guideline consensus

The question of whom to test has moved from selective screening toward near-universal, once-in-a-lifetime measurement, and the major guidelines now largely agree. Early recommendations were narrow. The first European Atherosclerosis Society consensus advised measuring Lp(a) in people at intermediate or high cardiovascular risk, such as those with a family history of premature disease (Nordestgaard et al., 2010). The initial National Lipid Association statement framed testing around specific clinical indications and cut-points (Wilson et al., 2019). Later documents broadened this position markedly. The 2022 European Atherosclerosis Society consensus recommended measuring Lp(a) at least once in every adult to refine lifetime cardiovascular risk (Kronenberg et al., 2022). The 2024 National Lipid Association update echoed this, advocating universal one-time testing (Koschinsky et al., 2024). Society guidelines outside dedicated lipid statements have aligned. The 2019 ESC/EAS dyslipidemia guideline advised measuring Lp(a) at least once to identify people with extreme genetically determined risk (Mach et al., 2019), and the 2018 AHA/ACC cholesterol guideline incorporated Lp(a) as a risk-enhancing factor to guide reclassification (Grundy et al., 2018). That document has since been retired. The 2026 ACC/AHA multisociety dyslipidemia guideline replaced it and is the first United States guideline to recommend measuring Lp(a) at least once in all adults for atherosclerotic cardiovascular disease risk assessment (Class 1, Level of Evidence B-NR), identifying ≥125 nmol/L or ≥50 mg/dL as a risk-enhancing concentration and reporting an approximately twofold estimated risk at ≥ 250 nmol/L (100 mg/dL) (Blumenthal et al., 2026). It further recommends cascade testing of first-degree relatives, notes that repeat testing may be considered for values in a threshold range, and requires that assays be insensitive to apo(a) isoform size and traceable to recognized reference materials, a requirement that ties the clinical recommendation directly to the analytical issues set out in Section 3.1. National frameworks such as the HEART UK consensus translated these principles into explicit testing indications and management pathways (Cegla et al., 2019). The biological rationale remains coherent: the predominantly genetic determination and general temporal stability of Lp(a) support one-time measurement for most adults, without implying that its concentration is biologically immutable. Table 1A summarizes guideline recommendations and risk thresholds, while Table 1B presents assay-specific analytical characteristics relevant to interpretation. The complete versions of these two tables are provided as Supplementary Tables S1, S2. Family history retains an independent role in deciding whom to test. Because Lp(a) concentration is largely determined at a single locus and transmitted in an essentially co-dominant fashion, an individual with markedly elevated Lp(a) identifies a family in which approximately half of first-degree relatives may also be affected. The 2024 National Lipid Association focused update accordingly recommends cascade screening of first-degree relatives of individuals with elevated Lp(a) (Koschinsky et al., 2024), an approach that parallels established practice in familial hypercholesterolaemia and that offers a practical route to case-finding in a population where opportunistic testing has demonstrably failed.

**TABLE 1A:** Guideline recommendations and risk thresholds for lipoprotein(a) measurement.

Guideline/statement (year)	Who to test	Risk categories/thresholds (units as printed)	Highest category/extreme-risk designation	Key position	Ref.
EAS consensus (2010)	Intermediate/high CV risk; premature CAD or family history	Desirable <80th percentile (<∼50 mg/dL), after LDL-C control	Not separately defined	Historical selective-testing framework	Nordestgaard et al., 2010
ESC/EAS dyslipidaemia guideline (2019)	Once in every adult’s lifetime	>50 mg/dL (∼125 nmol/L)	Extreme elevation: High/very-high risk without formal scoring	European once-in-a-lifetime testing	Mach et al., 2019
AHA/ACC cholesterol guideline (2018)	Selective use as a risk decision aid	≥50 mg/dL (≥125 nmol/L): Risk-enhancing	Not separately defined	Historical; replaced by the 2026 guideline	Grundy et al., 2018
NLA statement (2019)	Clinical indications or family history	≥50 mg/dL (≥100 nmol/L)	Not separately defined	Historical indication-based statement	Wilson et al., 2019
HEART UK consensus (2019)	Defined indications; no population-wide screening	32–90 nmol/L minor; 90–200 moderate; 200–400 high; >400 very high; management >90	>400 nmol/L (very high; >99.8th percentile)	UK testing and management pathway	Cegla et al., 2019
EAS consensus (2022)	Once in every adult	<30 mg/dL (<75 nmol/L): rule out; 30-50 (75–125): grey zone; ≥50 (≥125): rule in	No separate extreme category	Universal one-time testing; no fixed unit conversion	Kronenberg et al., 2022
NLA focused update (2024)	All adults once; cascade screening of first-degree relatives	<75 nmol/L (<30 mg/dL) low; 75-125 (30–50) intermediate; ≥125 (≥50) high	No separate extreme category	Graded bands; universal and cascade testing	Koschinsky et al., 2024
ACC/AHA multisociety dyslipidemia guideline (2026)	All adults once; cascade testing in affected families (COR 1, LOE B-NR)	≥125 nmol/L or ≥50 mg/dL: Risk-enhancing; ≥250 nmol/L (100 mg/dL): ∼2-fold risk	No separate category; ∼4-fold risk at 430 nmol/L (180 mg/dL)	First U.S. universal-testing guideline; isoform-insensitive, traceable assays	Blumenthal et al., 2026

Thresholds are reproduced in the units printed by each source; no fixed mass-to-molar conversion was applied. “Not separately defined” indicates no distinct extreme-risk cut-point. ASCVD, atherosclerotic cardiovascular disease; CAD, coronary artery disease; COR, class of recommendation; CV, cardiovascular; LDL-C, low-density lipoprotein cholesterol; LOE, level of evidence.

**TABLE 1B:** Assay-specific analytical characteristics relevant to lipoprotein(a) measurement.

Assay/platform	Measurand	Epitope	Calibration/traceability	Analytical range/dilution	LoQ/below-LoQ handling	Evidence of isoform independence	Ref.
KIV-2 repeat-binding direct ELISA (research assay)	Signal scales with KIV-2 repeats, not particle number	Repeated KIV-2 region	Serum calibrator; apo(a) with 21 KIV domains	Not reported	Not reported	None: Larger isoforms are overestimated and smaller isoforms underestimated versus the calibrator	Marcovina et al., 1995
LPA4/LPA-KIV9 monoclonal sandwich ELISA	Lp(a) particle number (nmol/L)	Detection: LETPTVV in single-copy KIV-9; capture interacts with KIV-2	WHO/IFCC SRM-2B (107 nmol/L)	0.27–4.38 nmol/L after 10-fold dilution; to 1,401 nmol/L with dilution up to 1:3,200; routine 1:400	LoQ 0.27 nmol/L; LOD and below-LoQ rule not reported	Bias versus KIV number: *r* ^2^ = 0.002 versus a-6/a-40 ELISA and 0.022 versus LC-MS/MS (n = 64)	Marcovina et al., 2022
Particle-enhanced turbidimetric assay, Roche c502	Reported in nmol/L; molecular species unspecified	Not reported	Not reported	Not reported	Not reported	Not stated in the trial report	Nissen et al., 2024b
LC-MRM-MS reference measurement procedure, IFCC-endorsed (semi-automated sample preparation; validated to ISO 15193)	Apo(a) via three proteotypic peptides; designed to be size-independent	Not applicable (peptide-based)	Five serum calibrators traceable to the former WHO/IFCC reference material SRM 2B; SI traceability not yet achieved	3.8–456 nmol/L	LoQ 3.8 nmol/L (LFLEPTQADIALLK); below-LoQ rule not reported	Peptides lie outside variable KIV-2; the procedure is IFCC-endorsed, and commutable ISO 15194-compliant serum-based secondary reference materials are now available	Ruhaak et al., 2023, van Neer et al., 2026, Cobbaert et al., 2026

Reporting unit alone does not establish isoform independence. 'Not reported’ indicates that the cited source did not state the parameter. ELISA, enzyme-linked immunosorbent assay; IFCC, international federation of clinical chemistry and laboratory medicine; KIV, kringle IV; LC-MRM-MS, liquid chromatography-multiple reaction monitoring-mass spectrometry; LOD, limit of detection; LoQ, limit of quantification; SI, international system of units; SRM, standard reference material.

### 3.3 Interpreting the value: cut-points, the intermediate range, and special populations

Once a value is available, its interpretation rests not on a single threshold but on a graded scale modified by clinical context. Because risk rises continuously across the distribution, any cut-point is a pragmatic simplification of a linear relationship (Patel et al., 2021). In practice, values below about 30 mg/dL are generally seen as adding little risk. Values above about 50 mg/dL (roughly 125 nmol/L) are risk-enhancing. The 30–50 mg/dL band is an intermediate zone, where risk should be weighed alongside other factors. Much higher values mark extreme, genetically determined risk. These thresholds are population-derived rather than individualized, and they are most useful when Lp(a) is integrated into broader risk assessment. Adding measured Lp(a), or a genetic risk score based on the *LPA* locus, to conventional models improves discrimination and reclassifies a meaningful fraction of people (Trinder et al., 2021).

Interpretation is further modified in specific populations. Chronic kidney disease and nephrotic-range proteinuria can raise Lp(a), so a value must be read in the light of renal function, not at face value (Hopewell et al., 2018). Hormonal status is a second modifier. Lp(a) tends to be higher after menopause and is lowered by hormone-replacement therapy, with oral estrogen producing the larger reduction. Both physiological state and concurrent therapy can thus shift serial measurements independently of cardiovascular risk (Anagnostis et al., 2017). The measured number is necessary but not self-interpreting.

### 3.4 The screening gap: from consensus to a stalled reality

Set against this converged guidance is a striking implementation failure: in the real world Lp(a) is almost never measured. An electronic health record analysis of a large regional health system found that Lp(a) was measured in fewer than one percent of patients undergoing lipid testing, and that rates were lower still among women and among Black and Hispanic or Latino patients, layering inequity onto underuse (Faridi et al., 2024). Broader assessments across health systems confirm persistently low testing rates and a wide gap between guideline recommendation and clinical practice (Bhatia et al., 2023). The drivers of this gap are mostly historical and structural, not scientific. For decades no specific therapy existed, which discouraged measurement of a factor that could not be modified. Laboratory ordering has rarely been made reflexive or automatic, and clinician awareness has lagged behind the evidence. The consequences are becoming more acute as targeted therapies approach the clinic. A population whose high Lp(a) is never measured cannot be offered screening-guided management, enrolled in outcomes trials, or considered for future therapy. The screening gap therefore threatens to blunt the clinical impact of the drugs discussed in the next section.

### 3.5 Progress, gaps, and open questions

Measurement and screening have advanced markedly, even as their clinical translation remains incomplete. On the analytical side, an internationally coordinated reference procedure and isoform-insensitive molar assays now offer, for the first time, a credible path to standardized reporting in nmol/L. On the clinical side, the guideline consensus around once-in-a-lifetime testing has removed most of the ambiguity about whom to test. The principal remaining gaps are practical. Global harmonization of units and assays is not yet complete, so mg/dL and nmol/L values still coexist. Population cut-points are not calibrated to ancestry, an equity concern taken up later in this review. And, most importantly, the once-in-a-lifetime recommendation has not been embedded in the systematic, reflexive testing pathways that would make it real. The key questions are correspondingly concrete: how to convert a broadly endorsed recommendation into automatic, system-level measurement; how to complete unit and assay harmonization so a value carries the same meaning everywhere; and how to individualize interpretation across the intermediate range and special populations. Answering them would settle the first of the two problems that run through this review: whether Lp(a) can be measured and interpreted reliably enough to act on in everyday practice. For the practicing cardiologist the operational message is simple. Measure Lp(a) once, interpret it in molar units and clinical context, and recognize that a reassuring mass-based value may still mislead. Acting on that measurement, though, requires knowing what to do once a high value is found, which is the subject of the next section.

## 4 Current management of cardiovascular risk in patients with elevated Lp(a)

In the absence of a proven Lp(a)-specific outcome therapy, the management of a patient with elevated Lp(a) is best understood not as a holding strategy but as the comprehensive reduction of that patient’s absolute and residual cardiovascular risk. This encompasses control of the total apoB-containing particle burden, blood pressure, smoking, metabolic dysfunction and diabetes, physical inactivity, obesity and adverse body composition, and renal disease, together with attention to thrombotic and inflammatory risk where clinically appropriate. Much of this will remain essential even if targeted agents prove effective, because an elevated Lp(a) identifies a patient whose lifetime risk warrants sustained attention across every modifiable domain, not merely the one for which a specific drug may become available.

Within that broader framework, the particle itself remains the one component that cannot yet be addressed on its own terms, because no approved drug lowers Lp(a) both substantially and specifically. Current practice combines aggressive control of conventional risk factors and intensive LDL-C lowering with, in selected refractory cases, lipoprotein apheresis. The lipid-lowering therapies in routine use affect Lp(a) only modestly and inconsistently. The evidence that any of these effects translates into event reduction comes mostly from *post hoc* analyses, not from trials designed to test the question. Setting out what these therapies do and do not achieve therefore serves two purposes: it defines the risk reduction that is deliverable today, and it clarifies why a specific, high-magnitude Lp(a)-lowering therapy has become such a clear unmet need.

The effect of established lipid-lowering drugs on Lp(a) ranges from a small increase to a moderate decrease, and even the strongest signals of benefit come from exploratory subgroup analyses. Statins, the cornerstone of LDL-C reduction, do not lower Lp(a) and on average modestly increase it. A meta-analysis of randomized trials reported mean increases of roughly 8%–20% (Tsimikas et al., 2020a). The response is heterogeneous, however, and individual changes vary in both magnitude and direction, so the group mean should not be read as a uniform effect. This paradox reinforces the need to measure Lp(a) independently of the LDL-C response. Ezetimibe is essentially neutral. PCSK9 inhibitors do lower Lp(a), typically by a quarter to a third. Analyses of the large outcomes trials suggest this reduction may add to clinical benefit. In FOURIER, patients with higher baseline Lp(a) gained greater absolute risk reduction from evolocumab (O'Donoghue et al., 2019). In ODYSSEY OUTCOMES, the Lp(a) reduction with alirocumab was associated with fewer major adverse cardiovascular events, partly independent of LDL-C (Bittner et al., 2020), with a parallel signal for peripheral arterial and venous thromboembolic events (Schwartz et al., 2020). These findings are biologically encouraging but must be read cautiously. They arise from *post hoc* subgroup analyses of trials not designed to test Lp(a) lowering, and cannot by themselves establish that reducing Lp(a) independently prevents events. Newer agents extend the same theme of modest effect. The small interfering RNA inclisiran, developed to lower LDL-C, produces only a limited reduction in Lp(a) (Ray et al., 2020). The oral cholesteryl ester transfer protein inhibitor obicetrapib lowers LDL-C by about 30% and lowers Lp(a) modestly as well (Nicholls et al., 2025a). Across this class, the reductions achieved are moderate and likely fall short of the large decrement that genetic modeling suggests would be needed to alter outcomes.

Against this background of partial pharmacological effects, lipoprotein apheresis is the intervention with the most suggestive clinical data. In a prospective observational study using a before-versus-after design, patients with elevated Lp(a) and progressive cardiovascular disease despite maximal drug therapy experienced markedly fewer major adverse cardiac events during apheresis than in the preceding period on medication alone (Leebmann et al., 2013). The inference that this benefit is attributable to Lp(a) specifically is limited by the uncontrolled design and by the fact that apheresis simultaneously lowers several apoB-containing lipoprotein fractions; the observation is therefore consistent with, but does not establish, an Lp(a)-specific effect on events. Its reach is also narrow. Apheresis is invasive, demanding of time and resources, and available under restricted indications. It therefore serves as a bridging therapy for a small refractory population, not a scalable solution. Randomized evidence has now begun to separate the Lp(a)-specific component of this effect from the rest of what apheresis removes. In the Lp(a)FRONTIERS APHERESIS trial, 51 patients already established on regular lipoprotein apheresis for elevated Lp(a) were randomly assigned, under quadruple masking, to pelacarsen 80 mg every 4 weeks or to placebo. Over 52 weeks the standardized rate of apheresis sessions fell to 0.16 with pelacarsen against 0.93 with placebo (odds ratio 0.006, 95% confidence interval 0.003–0.013). Two secondary endpoints should be distinguished. Apheresis avoidance was reached by 73.1% of pelacarsen-treated patients and by none receiving placebo, at a median of 6.1 weeks (hazard ratio 88.3, 95% confidence interval 4.71–1,653.15); separately, total apheresis avoidance from week 12 to week 52 was achieved by 69.2% versus none (odds ratio 163.2) (Parhofer et al., 2026). Because no placebo-treated patient reached either endpoint, these point estimates were derived under penalized likelihood and are correspondingly imprecise, as the very wide confidence interval indicates, although the direction of effect is unambiguous. Because the comparison is randomized and placebo-controlled, and because the intervention lowers Lp(a) selectively rather than depleting several lipoprotein fractions at once, this design isolates the contribution of Lp(a) in a way that the before-versus-after apheresis literature cannot. Its endpoint, however, is the need for apheresis rather than cardiovascular events, so it establishes Lp(a)-specific pharmacological substitution rather than Lp(a)-specific event reduction.

Two adjunctive strategies illustrate both the appeal and the limits of managing Lp(a) indirectly. Aspirin has drawn interest because genetic data suggest its benefit may be concentrated in people with high Lp(a). In the Women’s Health Study, carriers of an *LPA* variant gained a marked reduction in risk from aspirin that largely offset their excess risk (Chasman et al., 2009). In the ASPREE trial, older adults with a high-Lp(a) genotype had fewer cardiovascular events on aspirin, without a significant excess of major bleeding (Lacaze et al., 2022). These observations are hypothesis-generating rather than practice-changing, and do not yet justify aspirin specifically for high Lp(a) outside standard indications. The history of niacin is more instructive still. Niacin lowers Lp(a), and this effect once made it a plausible candidate. Yet in statin-treated patients neither AIM-HIGH nor HPS2-THRIVE showed any reduction in cardiovascular events, and the latter caused clear harm, definitively closing the niacin pathway (Boden et al., 2011; Landray et al., 2014). That a drug could lower Lp(a) and still fail to prevent events is an early and instructive observation, though it does not by itself isolate the reason for that failure. Insufficient absolute Lp(a) lowering, non-specific metabolic effects, adverse effects, and the intensity of background statin therapy are all plausible contributors, and these trials cannot separate them. The niacin experience is therefore consistent with the view that the magnitude and specificity of Lp(a) reduction matter, without demonstrating it. That theme is developed fully in the next section.

Taken together, the current options let clinicians reduce a patient’s global cardiovascular risk aggressively and touch Lp(a) modestly, but they offer no way to lower Lp(a) both specifically and substantially with proven outcome benefit. Intensive LDL-C control and comprehensive risk-factor management remain the foundation and will remain so whatever the outcome trials show, apheresis is reserved for extreme and refractory cases, and adjuncts such as aspirin remain unproven for this indication. The practical consequence is clear: a high Lp(a) value, once identified, still cannot be treated on its own terms. The failure of these indirect approaches also sharpens the second question of this review. The issue is no longer whether Lp(a) can be lowered but how large a reduction is needed to change outcomes. As the next section argues, the modest effects surveyed here almost certainly never reached that magnitude. This gap, between a causal, measurable risk factor and the absence of a specific, effective therapy, defines the unmet need that the emerging RNA-targeted and oral agents are designed to fill. It is to these therapies, and the outcomes trials now testing them, that the review turns next.

## 5 Targeted Lp(a)-lowering therapies and the CVOT era

For the first time, drugs designed specifically to lower lipoprotein(a) can reduce its concentration by about 65% to more than 95%, breaking the ceiling of magnitude that limited every earlier intervention. This capability transforms the therapeutic question. It is no longer whether Lp(a) can be lowered, but whether lowering it, and by how much, reduces cardiovascular events. That question remains open. Deep and durable reduction of a causal biomarker is a necessary condition for benefit, not proof of it. The chain of reasoning connecting a lower Lp(a) to fewer clinical events is exactly what the current generation of trials is built to test. This section follows the field in three steps. First, the mechanistic classes of targeted agents. Then their early-phase efficacy and the threshold hypothesis that defines how much lowering may be needed. Finally, the cardiovascular outcomes trials whose anticipated readouts will determine the standing of the whole field.

### 5.1 Mechanistic classes

Three mechanistic strategies now dominate targeted Lp(a) lowering, and all act by reducing the production or assembly of the particle rather than its clearance. The first uses an antisense oligonucleotide. Pelacarsen is a hepatocyte-directed, N-acetylgalactosamine-conjugated oligonucleotide that binds *LPA* messenger RNA and promotes its degradation, thereby suppressing hepatic apolipoprotein(a) synthesis (Tsimikas et al., 2020b). The second uses RNA interference. Small interfering RNAs such as olpasiran, lepodisiran, and zerlasiran harness the endogenous RNA-induced silencing complex to degrade *LPA* messenger RNA, and their catalytic action tends to produce deeper, more durable suppression than antisense chemistry (O'Donoghue et al., 2022; Nissen et al., 2023). The third departs from nucleic-acid chemistry altogether. Muvalaplin is an orally administered small molecule that disrupts the assembly of apolipoprotein(a) with apolipoprotein B, preventing formation of the Lp(a) particle rather than silencing its gene (Nicholls et al., 2023). Structural work has clarified how such a small molecule interferes with the specific apolipoprotein(a)–apolipoprotein B interaction, giving a mechanistic basis for oral inhibition (Diaz et al., 2024). These three modalities differ in route, dosing interval, and durability, but they share the same principle: shutting down particle production at its source. Table 2 summarizes these agents, their early-phase efficacy, and their cardiovascular outcomes trials.

**TABLE 2:** Targeted lipoprotein(a)-lowering agents: pharmacodynamic regimen, assay context, and cardiovascular outcomes development.

Agent/mechanism	Pharmacodynamic regimen	Representative Lp(a) response	Assay/measurand	Analytical reporting	Cardiovascular outcomes development	Ref
Pelacarsen GalNAc antisense oligonucleotide	Phase 2, SC: 20 mg q4w; 40 mg q4w; 60 mg q4w; 20 mg q2w; 20 mg weekly. Phase 3: 80 mg q4w	−35%; −56%; −72%; −58%; −80%, respectively. Mean within-group change at week 25 (q4w) or 27 (q2w/weekly); placebo: −6%	Isoform-independent Lp(a) assay; molar concentration (nmol/L). Platform, calibrator and traceabilityNR.	Analytical range, LoQ and below-LoQ handlingNR.	Lp(a)HORIZON (n = 8,323; established ASCVD) and Lp(a)FRONTIERS CAVS; results pending at literature cut-off	Tsimikas et al., 2020b; Novartis Pharmaceuticals, 2026a; Novartis Pharmaceuticals, 2026b
Olpasiran Small interfering RNA	SC, 75 mg q12w	−97.4% (95% CI −102.0 to −92.8); placebo-adjusted mean percent change at week 36. Placebo: +3.6%	Central-laboratory Lp(a), reported in nmol/L. Platform, calibrator and traceabilityNR.	Analytical range, LoQ and below-LoQ handlingNR.	OCEAN(a)-outcomes (n = 7,297; established ASCVD) and OCEAN(a)-PreEvent (primary prevention); results pending	O'Donoghue et al., 2022; Koren et al., 2022; Amgen, 2026a; Amgen, 2026b
Lepodisiran Long-duration small interfering RNA	SC, 400 mg on days 1 and 180; the two 400-mg groups were pooled for the primary analysis	−93.9% (95% CI −95.1 to −92.5); placebo-adjusted, time-averaged change over days 60–180. Only the day-1 dose contributed during this window	Central-laboratory serum Lp(a), reported in nmol/L. Platform, calibrator and traceabilityNR.	Analytical range, LoQ and below-LoQ handlingNR.	ACCLAIM-Lp(a) (estimated n = 17,300; established ASCVD or high risk of a first cardiovascular event); results pending	Nissen et al., 2023; Nissen et al., 2025; Eli Lilly and Company, 2026b
Zerlasiran (SLN360) Small interfering RNA	SC, 450 mg q24w (two doses)	−85.6% (95% CI −90.9 to −80.3), placebo-adjusted time-averaged change through week 36; median −94.5% (IQR −97.3 to −84.2) at week 36	Particle-enhanced turbidimetric assay, Roche c502; Lp(a) reported in nmol/L. Isoform independence and calibrator traceabilityNR.	Analytical range, LoQ and below-LoQ handlingNR.	No cardiovascular outcomes trial registered at literature cut-off	Nissen et al., 2022; Nissen et al., 2024a; Nissen et al., 2024b
Muvalaplin Oral apo(a)–apoB assembly inhibitor	Oral, 240 mg once daily	At week 12, placebo-adjusted reduction: −85.8% (95% CI −88.0 to −83.1) by intact-particle assay; −68.9% (95% CI −73.3 to −63.8) by apo(a)-based assay	Intact-particle assay: anti-apoB capture plus isoform-insensitive anti-apo(a) detection, measuring intact particles. Randox RX LP 3403: Total apo(a), including free or drug-bound apo(a)	Intact-assay vendor/platform, analytical range, LoQ and below-LoQ handling: NR; intact-assay data unavailable for 30 participants in China because sample storage was not permitted	MOVE-Lp(a) (estimated n = 10,450; established ASCVD or high risk of a first cardiovascular event); results pending	Nicholls et al., 2023; Diaz et al., 2024; Nicholls et al., 2025b; Eli Lilly and Company, 2026a

### 5.2 Drug-by-drug efficacy in early-phase trials

Across completed phase 1 and phase 2 studies, these agents have achieved deep reductions in Lp(a), while differing in the magnitude, onset, and persistence of effect. Pelacarsen opened the field. It produced dose-dependent reductions of roughly 35%–80% and showed that targeted suppression of apolipoprotein(a) synthesis was achievable (Tsimikas et al., 2020b). The small interfering RNAs have generally reached greater depth. Olpasiran lowered Lp(a) by more than 95% at higher doses in the phase 2 OCEAN(a)-DOSE study (O'Donoghue et al., 2022); earlier work showed that a single dose reduced concentrations by 71%–97% and sustained the effect for several months (Koren et al., 2022). Lepodisiran was engineered for still longer action. A single dose produced deep and durable reductions in phase 1 (Nissen et al., 2023), and the phase 2 ALPACA trial reported a placebo-adjusted, time-averaged reduction of about 94% at the 400-mg dose. This supports infrequent, broadly half-yearly, dosing as it advances into phase 3 (Nissen et al., 2025). Zerlasiran (formerly SLN360) produced deep reductions after a single dose in the APOLLO study (Nissen et al., 2022) and reproducible deep reductions across single and repeated dosing (Nissen et al., 2024a). In the phase 2 ALPACAR-360 trial the 450 mg every-24-week regimen achieved a placebo-adjusted, time-averaged reduction of 85.6% through 36 weeks; the median reduction at the week-36 timepoint itself was 94.5%, a reminder that time-averaged and single-timepoint metrics are not interchangeable (Nissen et al., 2024b). Finally, the oral inhibitor muvalaplin lowered Lp(a) by about 65% in first-in-human testing (Nicholls et al., 2023), showing that oral, non-injectable lowering is feasible. In the phase 2 KRAKEN study the 240 mg daily dose reduced Lp(a) at week 12 by 85.8% when measured with an assay that captures intact Lp(a) particles, but by 68.9% when measured with a conventional assay directed against apolipoprotein(a) (Nicholls et al., 2025b). The gap between these two figures is not measurement error but a direct consequence of the mechanism: because muvalaplin blocks the assembly of apolipoprotein(a) with apolipoprotein B rather than suppressing apolipoprotein(a) synthesis, apolipoprotein(a) continues to be produced and remains immunoreactive even though it is no longer incorporated into a particle. A conventional apolipoprotein(a) assay counts that residual protein and therefore understates the fall in intact Lp(a). The discrepancy is specific to assembly inhibition; for the messenger RNA–targeted agents, apolipoprotein(a) synthesis is itself suppressed and the two assay types agree more closely. Muvalaplin has now entered outcomes testing in MOVE-Lp(a) (NCT07157774) (Eli Lilly and Company, 2026a).

### 5.3 Comparing agents, and their safety profile

Because the agents differ in mechanism, route of administration, dosing convenience and durability rather than in their capacity to lower Lp(a) deeply, the practical distinctions concern administration and tolerability. Dosing intervals span a wide range. They run from the monthly injection typical of the antisense approach, through the quarterly or roughly half-yearly schedules of the longer-acting small interfering RNAs, to the daily oral dosing of muvalaplin. A network meta-analysis has begun to rank these agents by magnitude and durability of effect. Such cross-trial ranking is inevitably limited by heterogeneous designs, however, and should be read as provisional (Hu et al., 2026). Tolerability has so far been reassuring. The injectable agents are associated mainly with injection-site reactions, and no consistent serious safety signal has emerged across the class. Overall the early safety profile supports continued development, but definitive safety characterization, like definitive efficacy, awaits the outcome studies.

### 5.4 How much lowering is enough? The threshold hypothesis

The decisive scientific question is not whether these drugs lower Lp(a) but whether the reduction they achieve is large enough to matter, and genetic modeling offers the sharpest available guide. The relationship between Lp(a) and cardiovascular risk is continuous but relatively shallow on an absolute scale. So a small proportional change in a patient with modestly elevated Lp(a) may be clinically trivial, whereas a large absolute reduction in a patient with very high Lp(a) may not be. Mendelian analyses have tried to quantify the target. Their exact figures differ, but they agree that the required reduction is large. Both tie the estimate to the benefit of an approximately 1 mmol/L reduction in LDL-C. One places the equivalent Lp(a) reduction at roughly 100 mg/dL (Burgess et al., 2018), the estimate introduced in Section 2.3. A later analysis, revisiting the assumptions and the assay standardization involved, arrives at about 65 mg/dL and suggests that earlier estimates may have been too high (Lamina et al., 2019). The assumption that changed is the absolute Lp(a) concentration difference assigned to each genetic variant: the earlier analysis attributed larger per-variant effects on Lp(a), which distribute a given increment of coronary risk across a wider concentration difference and therefore imply a shallower risk gradient per 10 mg/dL and a correspondingly larger required reduction. The later authors attribute those larger per-variant effects in turn to the standardization of the Lp(a) assay from which the variant–concentration associations were derived, and conclude on that basis that the required reduction may previously have been overestimated. The point estimates are 101.5 mg/dL (95% CI, 71.0–137.0) and 65.7 mg/dL (95% CI, 46.3–88.3), intervals wide enough to overlap substantially. Both, moreover, describe lifelong genetic exposure rather than pharmacological intervention; the same reanalysis notes that the risk reduction associated with a genetically determined difference in LDL-cholesterol is roughly twice that observed in trials of LDL-lowering therapy, which cautions against reading either figure as a treatment threshold. The two values differ in magnitude rather than in the benchmark they use, and both imply that only a large absolute reduction is likely to matter. This threshold framing does real explanatory work. It offers a coherent explanation for the failure of earlier, weaker interventions, including niacin and the modest reductions seen with PCSK9 inhibition, whose effects on Lp(a) were probably too small in absolute terms to alter outcomes, although it is not the only explanation available for any one of them. It also explains why the deep reductions achieved by targeted agents may be necessary rather than merely impressive. The threshold hypothesis, though, remains a hypothesis. It predicts, from genetics, the magnitude of lowering likely to be needed, but it cannot by itself show that pharmacologically achieved lowering will prevent events. That demonstration requires randomized outcome data (Figure 3).

**FIGURE 3:**
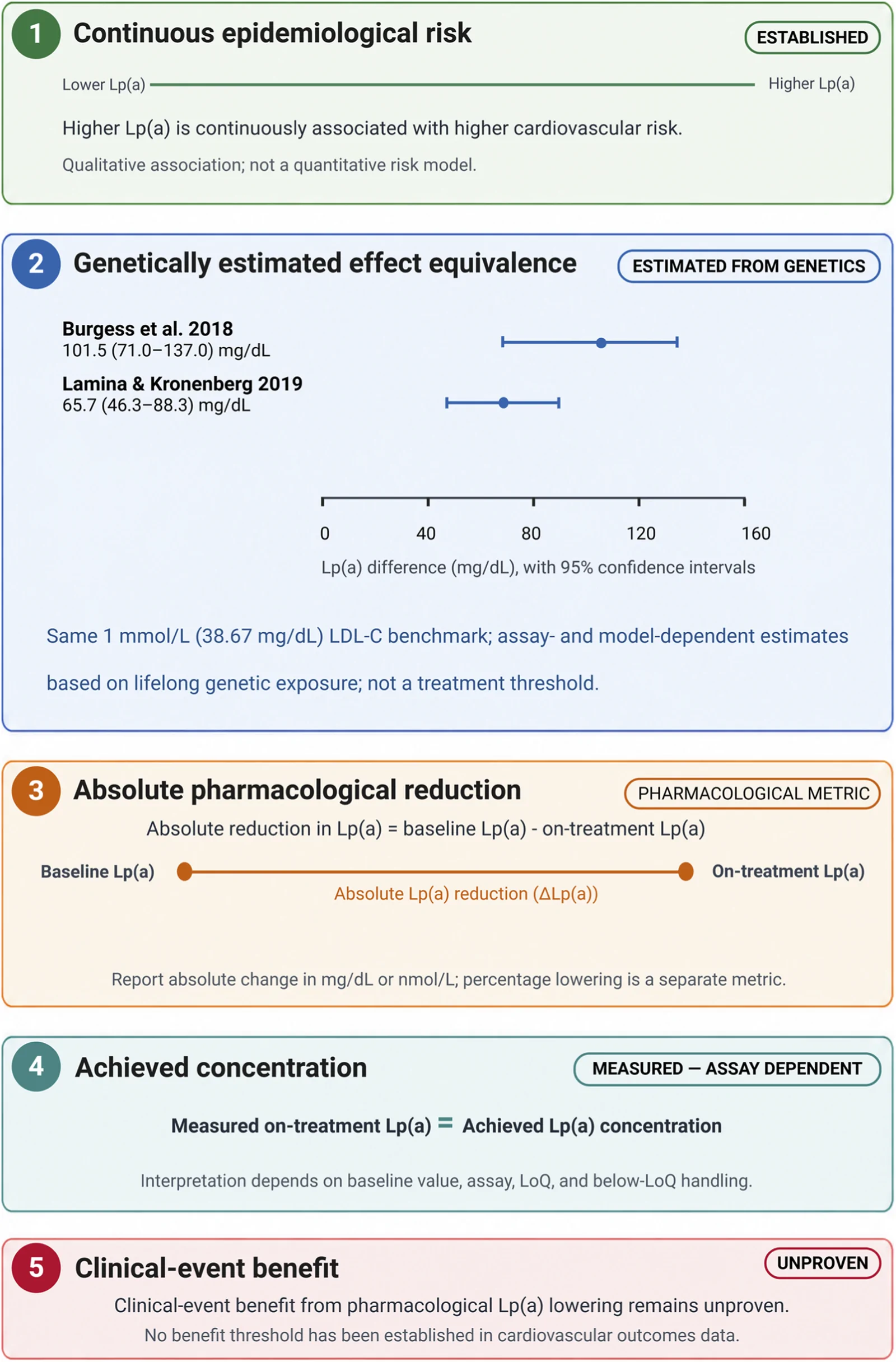
Layers of evidence underlying the magnitude question in lipoprotein(a) lowering. The panel separates five constructs that are frequently conflated and presents them from top to bottom as distinct, non-causal evidentiary and measurement layers. (1) The epidemiological association between Lp(a) concentration and cardiovascular risk is continuous and graded; the axis is deliberately unscaled, because this layer is not a quantitative risk model and no threshold has been identified in outcome data. (2) Mendelian effect-equivalence estimates express the Lp(a) difference whose association with coronary risk matches that of a 38.67 mg/dL (1 mmol/L) lower LDL-cholesterol concentration; both published estimates use this same reference benefit and are shown as point estimates with their 95% confidence intervals, the difference between them arising from the Lp(a) concentration estimates and assay architecture used rather than from the benchmark. The concentration scale in this layer is the only quantitative axis in the figure and applies to these two published estimates alone. Both derive from lifelong genetic exposure and are not equivalent to a pharmacological treatment target. (3) The absolute pharmacological reduction is the difference between the baseline and the on-treatment concentration and should be reported in mg/dL or nmol/L; proportional lowering is a separate metric and the two are not interchangeable. (4) The achieved concentration is the value actually measured on treatment, and its interpretation depends on the baseline concentration, the assay used, its limit of quantification and the handling of values below that limit. (5) Whether any of this translates into a reduction in clinical events is unproven; no cardiovascular outcomes trial has yet reported, and no benefit threshold has been established in outcome data.

### 5.5 The cardiovascular outcomes trial era

Several large cardiovascular outcomes trials are now testing the threshold hypothesis directly, and together their designs span the clinical questions that will define the field. Lp(a)HORIZON is evaluating pelacarsen in patients with established cardiovascular disease and elevated Lp(a). It is the first phase 3 trial to ask whether lowering Lp(a) reduces recurrent events in secondary prevention (Novartis Pharmaceuticals, 2026a). OCEAN(a)-Outcomes is testing olpasiran in a comparable high-risk secondary-prevention population (Amgen, 2026a), while ACCLAIM-Lp(a) is assessing lepodisiran in an estimated 17,300 participants with established ASCVD or at risk of a first cardiovascular event, exploiting its infrequent dosing (Eli Lilly and Company, 2026b). OCEAN(a)-PreEvent more specifically extends the question into a dedicated primary-prevention population (Amgen, 2026b). A separate line of work addresses the valve. Lp(a)FRONTIERS CAVS is testing whether pelacarsen slows the progression of calcific aortic-valve stenosis, the non-atherosclerotic disease for which Lp(a) is also causal (Novartis Pharmaceuticals, 2026b). Together these trials will answer several questions at once: whether lowering Lp(a) reduces atherosclerotic events, how much reduction is needed, whether benefit extends to primary prevention and to aortic-valve disease, and how the remaining safety questions resolve at scale.

The trajectory of this field over the coming years therefore depends less on further gains in potency, which appear largely achieved, than on the readouts of these outcome studies. The main progress is unambiguous: the magnitude ceiling has been broken, several agents lower Lp(a) deeply and durably, and oral administration is now feasible. The main gap is equally clear. Not one of these reductions has yet been shown to prevent a cardiovascular event, so the causal chain from biomarker to outcome remains open at its final link. The key questions the trials will resolve are how much lowering translates into benefit, in which populations that benefit is greatest, and whether the valve responds as the artery may. For the clinician, the practical implication is a period of watchful, cautious anticipation. A high Lp(a) should still be managed today as a risk-enhancing factor through global risk reduction. But the anticipated trial results may, for the first time, convert it from an unmodifiable marker into a directly actionable target. The broader implications of that prospect for practice, equity, and guidelines are considered in the final section.

Representative pharmacodynamic estimates are paired with the studied dose, statistical metric, time point, assay/measurand, and below-LoQ reporting available in the cited primary source. Values are not directly comparable across studies. NR indicates not reported in the cited primary publication; LoQ, limit of quantification; q2w, every 2 weeks; q4w, every 4 weeks; q12w, every 12 weeks; q24w, every 24 weeks; SC, subcutaneous. Trial status is current through 8 August 2026. No completed cardiovascular outcomes trial has yet shown that Lp(a)-specific lowering reduces cardiovascular events. Expanded dosing, analytical, and trial-design details are provided in Supplementary Table S3.

## 6 Challenges, future directions, and conclusion

Even before the outcomes trials report, several challenges will shape how lipoprotein(a) is used in practice, and they are as much about health systems as about biology. Where in the spectrum of prevention should targeted therapy be deployed? Can measurement and treatment be made cost-effective and equitable? How will trial readouts be translated into guidelines and clinical pathways? A positive trial result alone will not answer these questions. Beyond the current agents, more radical approaches such as gene editing are starting to appear on the horizon. Addressing these issues is what will finally close the clinical loop that this review has traced, from screening through stratification and management to targeted therapy.

The first challenge is positioning within the prevention spectrum. The ongoing trials concentrate on secondary prevention, and it is here that targeted therapy is most likely to enter practice first, because the high absolute event rates of established disease favor a manageable number needed to treat. Extending treatment to primary prevention is more demanding. In people without established disease the absolute benefit of lowering Lp(a) is smaller and slower to accrue, which raises the bar for both efficacy and long-term safety. Economic analyses have begun to map this terrain. Early health-technology assessment suggests that treating high Lp(a) in secondary prevention could be cost-effective under plausible assumptions (Burvill et al., 2024), and modeling indicates that even the upstream step of testing for Lp(a) may be cost-effective in primary prevention (Morton et al., 2025). These analyses reinforce the argument that a single, once-in-a-lifetime measurement is a rational foundation for a lifetime of risk-stratified decisions (Marcovina and Shapiro, 2022). Their conclusions, though, remain contingent on drug pricing and on the outcome data still to come.

A second set of challenges concerns equity and the biological diversity of Lp(a) itself. The distribution of concentrations and the strength of their link with cardiovascular risk vary substantially by genetic ancestry, so cut-points derived mainly from populations of European descent may misclassify risk in others (Guan et al., 2015). This variation is not a minor calibration issue but a recurring theme across ethnically diverse cohorts, in which both median concentrations and risk gradients differ (Reyes-Soffer, 2021). Whether it should translate into ancestry-specific cut-points remains debated. Some evidence favors ancestry-calibrated thresholds, while other analyses conclude that the predictive value of Lp(a) does not vary enough to mandate them (Tsimikas and Marcovina, 2022). At the same time the clinical scope of Lp(a) is widening beyond the coronary and valvular disease that have anchored most trials. Growing evidence links high concentrations to peripheral arterial disease, abdominal aortic aneurysm, and adverse limb events, each a possible future target for measurement and therapy (Thomas et al., 2023). Looking further ahead, genome-editing strategies that disrupt the *LPA* gene could achieve near-complete and potentially permanent elimination of apolipoprotein(a). They have provided proof of concept in preclinical work, hinting at a one-time intervention that would sit beyond even the long-acting RNA agents (Doerfler et al., 2022). Such an approach would, however, be irreversible, and the long-term physiological consequences of the near-complete and permanent absence of apolipoprotein(a) in humans are unknown; the plasminogen homology of apo(a) and its uncertain physiological role make this uncertainty difficult to resolve in advance of long-term human data. This uncertainty attaches specifically to near-total and permanent elimination, and not to the exposure produced by lowering an elevated concentration toward the population median, which is what the current agents and their outcome trials are testing.

The final challenge is implementation: turning evidence into everyday practice. The persistent gap between guideline recommendation and real-world testing is already the central weakness of current screening. It will only become more consequential once effective therapies exist, because an untested population cannot be treated (Bhatia et al., 2023). Bridging this gap will require practical, accessible clinical guidance, of the kind that consensus groups have begun to provide in question-and-answer form for front-line clinicians (Kronenberg et al., 2023). It may also require coordinated policy action. Recent international declarations calling for harmonized testing and management standards reflect a growing recognition that the barriers are systemic, not merely clinical (Kronenberg et al., 2025). In conclusion, within a single generation lipoprotein(a) has moved from a causally implicated but untreatable risk factor to one of the most important residual-risk targets of the statin era. This review has followed it through a continuous clinical and pharmacological loop: from standardized measurement and once-in-a-lifetime screening, through risk stratification and the global risk reduction that constitutes current management, to the targeted therapies capable of near-complete lowering. The final link in that loop, the demonstration that lowering Lp(a) prevents events, is not yet in place, and the anticipated cardiovascular outcomes trials are the inflection point at which it may be forged. Whatever their result, integrating accurate measurement, equitable screening, and mechanism-based therapy into a coherent clinical pathway is the task that will let the field act decisively on one of the most common and long-neglected causes of cardiovascular risk.
