# Supplementary material for: Lipoprotein(a) in the statin era: from risk marker to therapeutic target
Source: Front Pharmacol. 2026 Sep 16;17:1938864. doi: 10.3389/fphar.2026.1938864 (PMC13623574; doi:10.3389/fphar.2026.1938864)
Supplement: Supplementary file 1 [file Table1.pdf]

Supplementary Tables

Lipoprotein(a) in the statin era: from risk marker to therapeutic target

Supplementary Table S1. Complete guideline recommendations and risk thresholds for Lp(a) measurement.

Guideline / statement (year)	Who to test	Risk categories / thresholds (units as printed in the source)	Highest category / extreme-risk designation	Key position	Ref
EAS consensus (2010)	Patients at intermediate or high CV risk; premature CAD or family history	Desirable level < 80th percentile (< ~50 mg/dL), as a secondary priority after LDL-C lowering	Not separately defined	First consensus to recommend selective Lp(a) screening in high-risk individuals; its evidence grading rests on meta-analysis of niacin trials rather than on Lp(a) lowering itself	Nordestgaard et al., 2010
ESC/EAS dyslipidaemia guideline (2019)	At least once in every adult's lifetime	> 50 mg/dL (~125 nmol/L)	Extreme elevation treated as a high- or very-high-risk state without formal risk scoring	Established once-in-a-lifetime measurement in European practice	Mach et al., 2019
AHA/ACC cholesterol guideline (2018)	Selective use as a risk decision aid	≥ 50 mg/dL (≥ 125 nmol/L) as a risk-enhancing factor	Not separately defined	Lp(a) listed as a risk-enhancing factor to guide statin reclassification; retired and replaced by the 2026 multisociety guideline	Grundy et al., 2018
NLA statement (2019)	Testing by clinical indication / family history	≥ 50 mg/dL (≥ 100 nmol/L)	Not separately defined	Indication- and cut-point-based use of Lp(a) in clinical practice	Wilson et al., 2019
HEART UK consensus (2019)	Defined testing indications and management pathway; does not endorse population-wide screening	32–90 nmol/L minor; 90–200 nmol/L moderate; 200–400 nmol/L high; > 400 nmol/L very high; management threshold > 90 nmol/L	> 400 nmol/L (very high; > 99.8th percentile)	National framework translating principles into explicit indications and pathways	Cegla et al., 2019
EAS consensus (2022)	At least once in every adult	Pragmatic bands: rule out < 30 mg/dL (< 75 nmol/L); interim grey zone 30–50 mg/dL (75–125 nmol/L); rule in ≥ 50 mg/dL (≥ 125 nmol/L)	Not defined as a category within the rule-in / rule-out framework	Universal once-in-a-lifetime testing to refine lifetime CV risk; declines to endorse a fixed mass-to-molar conversion factor	Kronenberg et al., 2022
NLA focused update (2024)	Universal one-time testing in all adults; cascade screening of first-degree relatives of individuals with elevated Lp(a)	< 75 nmol/L (< 30 mg/dL) low; 75–125 nmol/L (30–50 mg/dL) intermediate; ≥ 125 nmol/L (≥ 50 mg/dL) high	Not separately defined; the update calls for retirement of a single dichotomous threshold	Reaffirms and broadens universal one-time measurement; replaces a dichotomous cut-point with graded bands and adds cascade screening	Koschinsky et al., 2024
ACC/AHA multisociety dyslipidemia guideline (2026)	All adults, at least once, for ASCVD risk assessment (COR 1, LOE B-NR); cascade testing of first-degree relatives where familial hypercholesterolaemia, premature ASCVD, or high Lp(a) is present (COR 1, LOE B-NR)	≥ 125 nmol/L or ≥ 50 mg/dL as a risk-enhancing factor; ≥ 250 nmol/L (100 mg/dL) associated with approximately twofold ASCVD risk relative to the population median	No separate extreme category; the accompanying risk table reports approximately fourfold risk at 430 nmol/L (180 mg/dL)	First US guideline to recommend measurement in all adults; retires and replaces the 2018 document; requires assays insensitive to apo(a) isoforms and traceable to official reference standard materials (COR 1, LOE B-NR)	Blumenthal et al., 2026

Thresholds are reproduced in the units printed by each source. No fixed mass-to-molar conversion was applied, because apo(a) isoform heterogeneity precludes a universal conversion factor. “Not separately defined” indicates that the source does not specify a distinct extreme-risk cut-point. ASCVD, atherosclerotic cardiovascular disease; COR, class of recommendation; LOE, level of evidence; CAD, coronary artery disease; CV, cardiovascular; FH, familial hypercholesterolaemia.

Supplementary Table S2. Assay-specific analytical characteristics relevant to Lp(a) measurement.

Assay / platform	Measurand	Epitope	Calibration hierarchy and traceability	Analytical range / dilution protocol	LoQ and below-LoQ handling	Evidence of isoform independence	Ref
Direct-binding ELISA with a detection antibody specific for the apo(a) kringle IV type-2 repeats (research assay)	Signal proportional to the number of KIV-2 repeats carried by the particle rather than to the number of particles	Within the repeated KIV-2 region	Calibrated against a serum whose apo(a) carried 21 kringle IV domains	Not reported	Not reported	None. Lp(a) is overestimated when apo(a) is larger than the calibrator (> 21 kringle IV domains) and underestimated when it is smaller. In the same study, two assays whose detection antibodies did not recognise the KIV-2 repeats gave essentially identical, size-independent results, which locates the bias in the epitope rather than in the reporting unit.	Marcovina et al., 1995
Monoclonal antibody sandwich ELISA (LPA4 capture, LPA-KIV9 detection). This is one specific assay, not a generic class of molar assays.	Number of circulating Lp(a) particles, reported in nmol/L; explicitly not the variable mass of the particle	Detection antibody binds LETPTVV in KIV-9, present in a single copy on apo(a). The capture antibody does interact with KIV-2, so isoform independence rests on the single-copy detection epitope rather than on the assay format.	WHO/IFCC first reference material for Lp(a) immunoassays, SRM-2B, assigned value 107 nmol/L, used as the assay calibrator	0.27 to 4.38 nmol/L after the 10-fold minimum required dilution, extended to 0.27 to 1,401 nmol/L using dilutions up to 1:3,200; routine sample dilution 1:400	Lower limit of quantification 0.27 nmol/L after the minimum required dilution. Limit of detection not reported, and no rule for handling below-limit values is stated.	In 64 samples of known apo(a) isoform size, bias versus kringle IV number gave $r^2 = 0.002$ against the a-6/a-40 ELISA and $r^2 = 0.022$ against LC-MS/MS, so apo(a) size explained 0.2% and 2.2% of the variation in bias. The number of distinct isoform sizes covered is not reported.	Marcovina et al., 2022
Particle-enhanced turbidimetric assay on a Roche c502 clinical analyser, as used to generate phase 2 pharmacodynamic data	Reported in nmol/L; the molecular species measured is not specified	Not reported	Not reported	Not reported	Not reported	Not stated by the trial report	Nissen et al., 2024b
LC-MRM-MS candidate reference measurement procedure, provisionally validated to ISO 15193	Apo(a) protein, quantified through three proteotypic peptides (LFLEPTQADIALLK, GISSTVTGR, TPENYPNAGLTR), by design independent of apo(a) size polymorphism	Not applicable: peptide-based rather than antibody-based	A single human serum calibrator traceable to the former WHO/IFCC reference measurement system. The procedure is intended as part of a future SI-traceable chain rather than as an achieved SI-traceable result.	Linear from 3.8 to 456 nmol/L	Lower limit of quantification 3.8 nmol/L using peptide LFLEPTQADIALLK; no rule for handling below-limit values is stated.	Achieved by design, through peptides lying outside the variable KIV-2 repeat. Formal endorsement as a reference measurement procedure has not been reported, and commutable secondary reference materials remain under development.	Ruhaak et al., 2023

Assays are organized by measurand, antibody epitope, calibration, analytical range, limit of quantification, and evidence of apo(a)-isoform independence. Reporting unit alone does not establish isoform independence. “Not reported” means the cited source does not state the parameter. ELISA, enzyme-linked immunosorbent assay; IFCC, International Federation of Clinical Chemistry and Laboratory Medicine; KIV, kringle IV; LC-MRM-MS, liquid chromatography-multiple reaction monitoring mass spectrometry; LoQ, limit of quantification; SRM, standard reference material.

Supplementary Table S3. Detailed targeted Lp(a)-lowering agents, efficacy metrics, assay information, and outcomes trials.

Agent	Mechanistic class	Route, dose and schedule	Lp(a) reduction	Metric and timepoint	Assay and measurand	LoQ / below-LoQ handling	Cardiovascular outcomes trial (NCT, n, population)	Ref
Pelacarsen	Antisense oligonucleotide (GalNAc-conjugated, anti-LPA mRNA)	Subcutaneous. Phase 2: 20 mg q4w; 40 mg q4w; 60 mg q4w; 20 mg q2w; 20 mg weekly. The phase 3 programme uses 80 mg q4w.	−35%; −56%; −72%; −58%; −80%, respectively	Mean percent change from baseline, not placebo-adjusted; week 25 for the every-4-week regimens and week 27 for the every-2-week and weekly regimens. The placebo group changed by −6%.	Isoform-independent assay reporting molar concentration (nmol/L); platform, calibrator and traceability not specified in the primary report	Not reported in the primary publication	Lp(a)HORIZON (NCT04023552); n = 8,323; established ASCVD, entry Lp(a) ≥ 70 mg/dL, with co-primary endpoints in the ≥ 70 and ≥ 90 mg/dL populations; primary completion June 2026, not yet reported. Lp(a)FRONTIERS CAVS (NCT05646381), calcific aortic-valve stenosis.	Tsimikas et al., 2020b; Novartis Pharmaceuticals, 2026a; Novartis Pharmaceuticals, 2026b
Olpasiran	Small interfering RNA	Subcutaneous, 75 mg q12w (phase 2 also tested 10 mg and 225 mg q12w, and 225 mg q24w)	−97.4% (95% CI −102.0 to −92.8)	Placebo-adjusted mean percent change from baseline at week 36. Placebo-adjusted values approach or exceed 100% because the placebo group rose by 3.6%; the corresponding within-group change was −93.8%.	Central-laboratory assay reporting nmol/L; the assay is not identified in the primary report	Not reported in the primary publication	OCEAN(a)-Outcomes (NCT05581303); n = 7,297; established ASCVD, entry Lp(a) ≥ 200 nmol/L; primary completion March 2028. OCEAN(a)-PreEvent (NCT07136012), primary prevention.	O'Donoghue et al., 2022; Koren et al., 2022; Amgen, 2026a; Amgen, 2026b
Lepodisiran	Small interfering RNA (long-duration)	Subcutaneous, 400 mg on day 1 and day 180; the two 400 mg arms were pooled (n = 141) because dosing was identical through day 180	−93.9% (95% CI −95.1 to −92.5)	Placebo-adjusted, time-averaged percent change from baseline over days 60 to 180. Only the day-1 dose was on board during that window.	Serum Lp(a) in nmol/L at a central laboratory; the assay is not identified in the primary report	Not reported in the primary publication	ACCLAIM-Lp(a) (NCT06292013); estimated n = 17,300; established ASCVD or at risk of a first cardiovascular event; entry Lp(a) ≥ 175 nmol/L; primary completion March 2029	Nissen et al., 2023; Nissen et al., 2025; Eli Lilly and Company, 2026b
Zerlasiran (SLN360)	Small interfering RNA	Subcutaneous, 450 mg q24w (two doses); phase 2 also tested 300 mg q16w and 300 mg q24w	−85.6% (95% CI −90.9 to −80.3)	Placebo-adjusted, time-averaged percent change from baseline through week 36. The single-timepoint median change at week 36 in the same arm was −94.5% (IQR −97.3 to −84.2); the two metrics are not interchangeable.	Particle-enhanced turbidimetric assay on a Roche c502 analyser, reported in nmol/L; isoform independence and calibrator traceability are not stated	Not reported in the primary publication	None registered	Nissen et al., 2022; Nissen et al., 2024a; Nissen et al., 2024b
Muvalaplin	Oral small molecule (blocks apo(a)–apoB particle assembly)	Oral, 240 mg once daily	−85.8% (95% CI −88.0 to −83.1)	Placebo-adjusted percent change from baseline at week 12	Intact Lp(a) particle assay: sandwich format combining an anti-apoB capture antibody with an isoform-insensitive anti-apo(a) detection antibody; measures intact Lp(a) particles. Vendor and platform are not named, and the assay is not available in clinical practice.	Not reported. Intact-assay data were unavailable for 30 participants in China because sample storage was not permitted.	MOVE-Lp(a) (NCT07157774); estimated n = 10,450; established ASCVD or at risk of a first cardiovascular event; entry Lp(a) ≥ 175 nmol/L; primary completion March 2031	Nicholls et al., 2023; Diaz et al., 2024; Nicholls et al., 2025b; Eli Lilly and

Agent	Mechanistic class	Route, dose and schedule	Lp(a) reduction	Metric and timepoint	Assay and measurand	LoQ / below-LoQ handling	Cardiovascular outcomes trial (NCT, n, population)	Ref
								Company, 2026a
Muvalaplin (same participants and dose, second measurand)	—	Oral, 240 mg once daily	−68.9% (95% CI −73.3 to −63.8)	Placebo-adjusted percent change from baseline at week 12. The response on this assay is non-monotonic: 60 mg gave −70.0%, exceeding the 240 mg estimate.	Randox RX series LP 3403: polyclonal anti-apo(a) antibodies measuring the total molar concentration of apo(a), including apo(a) that is free or drug-bound and therefore not part of an intact particle	Not reported in the primary publication	As above	Nicholls et al., 2023; Diaz et al., 2024; Nicholls et al., 2025b; Eli Lilly and Company, 2026a

Every estimate is reported with the metric on which it is based, because peak, trough, time-averaged and placebo-adjusted values are not interchangeable and cross-agent comparison is therefore approximate at best. Two consequences of this deserve emphasis. First, the pelacarsen phase 2 values are within-group changes from baseline rather than placebo-adjusted estimates, so they are not directly comparable with the placebo-adjusted values reported for the other agents. Second, the two muvalaplin rows are not alternative estimates of one quantity: they measure different molecular species, and the difference between them is mechanistically expected, because muvalaplin blocks apo(a)–apoB assembly without suppressing apo(a) synthesis, leaving apo(a) that remains immunoreactive without forming an intact particle. This divergence is specific to the assembly inhibitor; for the mRNA-targeted agents, apo(a) synthesis itself is suppressed and the two assay types are more nearly concordant. No primary publication for any of these agents reports a limit of quantification or a rule for handling below-limit values, which is itself an illustration of the standardization gap described in Section 3.1. Indirect network meta-analytic ranking of these agents is provisional (76). No completed cardiovascular outcomes trial has yet shown that lowering Lp(a) reduces cardiovascular events; all outcome trials remain ongoing. A possible glycaemic signal observed with olpasiran is discussed in Section 5.3. ASCVD, atherosclerotic cardiovascular disease; CI, confidence interval; IQR, interquartile range; LoQ, limit of quantification; q2w/q4w/q12w/q16w/q24w, every 2/4/12/16/24 weeks.
